# Predictive model for the therapeutic effect of bilateral subthalamic nucleus deep brain stimulation on the freezing of gait in Parkinson’s disease

**DOI:** 10.3389/fnagi.2025.1511845

**Published:** 2025-05-27

**Authors:** Qi Limuge, Yuqing Zhang, Xiaofei Jia, Fang Wang, Dongsheng Yu, Lixia Chen, Li Zhang, Yongqiang Jiang

**Affiliations:** ^1^Department of Neurosurgery, Baotou Central Hospital, Affiliated Baotou Clinical College of Inner Mongolia Medical University, Baotou, China; ^2^Department of Functional Neurosurgery, Xuanwu Hospital, Capital Medical University, Beijing, China; ^3^Inner Mongolia People’s Hospital, Hohhot, China; ^4^Department of Neurosurgery, Inner Mongolia Third Hospital, Hohhot, China

**Keywords:** Parkinson’s disease, freezing of gait, prediction model, deep brain stimulation, subthalamic nucleus

## Abstract

**Background:**

Freezing of gait (FOG) is a major disabling symptom that affects the quality of life of patients with Parkinson’s disease (PD). To date, notions regarding the effects of deep brain stimulation of the subthalamic neucleus (STN-DBS) on FOG remain controversial. Therefore, we developed a prediction model based on the influence of bilateral deep brain stimulation (DBS) of the subthalamic nucleus (STN) on FOG in patients with PD.

**Methods:**

We collected data from 104 PD participants with FOG who underwent STN-DBS at Xuanwu Hospital between September 2017 and June 2022. The patients were divided into a training set (70%; *n* = 68) and a validation set (30%; *n* = 36). The selected characteristics in the LASSO regression were used in multivariate logistic regression to build the prediction model. The receiver operating characteristic (ROC) curves were constructed for the training and validation sets to verify the model’s efficiency.

**Results:**

Independent variables in the prediction model included Unified Parkinson’s Disease Rating Scale II (UPDRS II), UPDRS IV, leg rigidity, Montreal Cognitive Assessment (MoCA) score, and Mini-Mental State Examination (MMSE) score. The prediction model formula is as follows: Logit(y) = −1.0043 + 0.159 × UPDRS II + 0.030 × UPDRS IV - 1.726 × leg rigidity + 0.121 × MoCA + 0.036 × MMSE. To validate the model, we analyzed the ROC curves of the training and validation sets. The area under the ROC curve (AUC) of internal validation was 0.869 (95% confidence interval [CI]: 0.771–0.967) and the AUC of external validation was 0.845 (95% CI: 0.6526–1). The calibration plots showed good calibration.

**Conclusion:**

The model we developed can effectively assist clinicians in assessing the efficacy of deep brain stimulation of the bilateral subthalamic nucleus for freezing of gait in Parkinson’s disease patients. This approach can support the formulation of personalized treatment plans and has the potential to improve patient outcomes.

## Introduction

Parkinson’s disease (PD) is a complex, chronic, and progressive neurodegenerative disease (Bloem et al., 2021). Freezing of gait (FOG) is a major motor disability that affects the daily quality of life of patients with PD. It usually occurs when starting, turning, or approaching a target. FOG can be induced or aggravated by narrow aisles or thresholds, crowded and noisy places, dual-task interference, time pressure, and psychological stress (Rahimpour et al., 2021). Statistics reveal that the incidence of FOG in the early stage of PD is 27%, while the incidence of FOG in the late stages of PD can reach 80% (Tan et al., 2011). From a neurophysiological viewpoint, the motor symptoms of PD appear to result largely from abnormalities in one of the several parallel and largely segregated basal ganglia thalamocortical circuits in the human brain (Bostan et al., 2013; Hallett, 2014). Dysfunction of one or more of these circuits—either individually or in combination—results in the disruption of downstream network activity in the thalamus, cortex, and brainstem (DeLong and Wichmann, 2015). Deep brain stimulation (DBS) involves implanting a stimulation electrode into a target nucleus deep within the brain tissue of the patient, followed by the delivery of weak electric pulses at specific frequencies via a pulse generator, allowing downstream network activity to function more normally (Fejeran et al., 2022). DBS in the subthalamic nucleus (STN) can alleviate tremors, rigidity, and dyskinesias and reduce the doses of medications, even among patients with advanced stages of the disease, which makes the STN a preferred target for DBS (Hariz and Blomstedt, 2022). At present, several studies have focused on the effect of bilateral STN-DBS on FOG, while others have revealed improved gait in the first few years following surgery, which gradually deteriorates with disease progression (Kim et al., 2019; Krack et al., 2003). This deteroitation is reminiscent of the reduced responsiveness of axial symptoms to L-dopa in the long term (Ferraye et al., 2008). Phibbs et al. (2014) propose that the improvement of axial symptoms, such as FOG, in DBS therapy is closely associated with maintaining a constant range of total electrical energy delivered (TEED). In the early postoperative period, the stimulation voltage is relatively low, enabling TEED to remain within a stable range, which effectively alleviates FOG and other axial symptoms. However, as Parkinson’s disease (PD) progresses, clinicians often gradually increase the stimulation voltage to address worsening symptoms. While this adjustment aims to improve motor symptoms, it may lead to an imbalance in TEED, thereby disrupting the stability of neural network regulation. This imbalance could further exacerbate the deterioration of FOG symptoms (Phibbs et al., 2014; Koss et al., 2005). Thus, the efficacy and effects of STN-DBS on FOG levels remain controversial.

This study investigated the clinical preoperative evaluation of patients with PD to further assess and discuss the effect of bilateral STN-DBS on FOG. Furthermore, for the first time, we developed a prediction model based on the influence of STN-DBS on FOG in patients with PD.

## Materials and methods

### Study population

This study investigated 104 patients with PD with FOG who underwent bilateral STN-DBS at the Xuanwu Hospital from September 2017 to June 2022.

The data used in this study were obtained from the Xuanwu Hospital and were completely anonymized prior to analysis. As no identifiable patient information was used, our institutional review board (IRB) granted a waiver of informed consent. The IRB reviewed and approved the study protocol, confirming that it met ethical standards for retrospective research.

### Inclusion and exclusion criteria

The inclusion criteria were as follows: (1) a definite diagnosis of primary PD, (2) a preoperative state without medicine (Med-off state) with a Movement Disorder Society (MDS)-Unified Parkinson’s Disease Rating Scale III (UPDRS III) item 11 freezing score ≥1, (3) bilateral STN-DBS performed, and (4) postoperative follow-up for 1 year. The exclusion criteria were as follows: (1) patients with secondary PD caused by cerebral infarction, cerebral hemorrhage, and drugs, among others, (2) patients with PD who could not complete the MDS-UPDRS III, and (3) patients with other brain tissue diseases or who had undergone other brain surgeries.

### Groups

A total of 104 participants were divided into training (70%; *n* = 68) and validation sets (30%; *n* = 36).

Participants in the training set were further divided into the improved (*n* = 58) and non-improved (*n* = 10) groups, while the validation set was also divided into the improved (*n* = 31) and non-improved (*n* = 5) groups. Grouping was based on the difference in the MDS-UPDRS III (Regnault et al., 2019) item 11 freezing score between the preoperative med-off status and the postoperative status at 1 year (Stim-on/Med off). Scores with differences of ≥1 and <1 were categorized as the improved and non-improved groups, respectively.

### Data collection

We collected 30 influencing factors, including general characteristics as well as motor and non-motor symptom scores.

1. The general information included sex, age, age at onset, disease duration, and preoperative levodopa-equivalent daily dose (LEDD).
2. The motor symptom assessment included the MDS-UPDRS II and III (including leg activity, leg rigidity, leg tremor, and postural stability), MDS-UPDRS IV, New Freezing of Gait Questionnaire (NFOGQ) (Mezzarobba et al., 2023), the improvement rate of the levodopa stress test, the Berg Balance Scale (BBS), the Hoehn and Yahr stage scale (HY-stage scale), and the Unified Dyskinesia Rating Scale (UDysRS).
3. The non-motor symptoms assessments included the Montreal Cognitive Assessment (MoCA) and Mini-Mental State Examination (MMSE) to assess cognitive status. The Hamilton Anxiety Scale (HAMA) and Hamilton Depression Scale (HAMD) were use to assess the patients’ moods, and the Epworth Sleepiness Scale (ESS), Pittsburgh Sleep Quality Index (PSQI), Rapid Eye Movement Sleep Disorder Questionnaire - Hong Kong edition (RBD-HK), and Non-motor Symptoms Scale (NMSS) and MDS-UPDRS I were used to assess other non-motor symptoms.

### Statistical analysis

The Statistical Package for the Social Sciences version 25.0 was used for descriptive data analysis. Measurement data conforming to normal distribution are expressed as mean ± standard deviation (x̄ ± s), and an independent sample *t*-test was used for comparison between the groups. The median and quartile [M (P25–P75)] represent the non-normally distributed measurement data, and the Mann–Whitney U-test was used for between-group comparisons. Counting data are expressed as a percentage (%), and the *X^2^* test was used for between-group comparison. The R4.3.2 “glmnet” package was used for the least absolute shrinkage and selection operator (LASSO) analysis to select variables before developing the predictive model to select the optimal variables and eliminate redundant ones. A multivariate logistic regression analysis was performed, and a visual nomogram was constructed as a predictive model based on the variables selected from the LASSO regression model. Receiver operating characteristic (ROC) (Nahm, 2022) curve analysis, calibration curve, and decision curve analysis (DCA) (Vickers and Holland, 2021) were used to assess the differentiation and calibration degree of the model. The ROC curve was generated for the validation set data, and the area under the ROC curve (AUC) values of the training and validation sets were compared to complete the external validation of the model.

## Results

### Comparison of clinical data between groups in the training set

Participants in the training set (68 participants in total) were categorized into the improved (58 participants) and non-improved (10 participants) groups. A comparison of clinical data between the improved and non-improved groups revealed that the median age (non-improved: 65.7 ± 7.96 vs. improved: 63.88 ± 7.56), MDS-UPDRS I (non-improved: 14.6 ± 5.97 vs. improved: 13.62 ± 5.72), and MDS-UPDRS III (non-improved: 62 ± 10.76 vs. improved: 61 ± 16.3) were significantly higher in the non-improved group than the improved group. Additionally, the median disease duration [non-improved: 9.5 (5.75–13.25) vs. improved: 9 (7–12.25)], NFOGQ score [non-improved: 25 (15.75–26.25) vs. improved: 24 (20–27)], leg rigidity [non-improved: 2.5 (2.375–3) vs. improved: 2 (1.5–2.5)], and HAMD [non-improved: 9.5 (6.5–11.75) vs. improved: 9 (5–13.25)] were also higher in the non-improved group compared to the improved group. A statistically significant difference in leg rigidity was observed between the two groups (*p* = 0.025 < 0.05). It should be noted that the non-improved group consisted of only 10 patients, which may limit the generalizability of our findings. Further studies with larger sample sizes are needed to confirm these results. (Table 1).

**Table 1:** Comparison of clinical data between the improved and non-improved groups in the training set.

Factor	Non-improved group	Improved group	*p*-value
Sex			0.701
Female	4 (40%)	27 (46.6%)	
Male	6 (60%)	31 (53.4%)	
Age (years)	65.7 ± 7.96	63.88 ± 7.56	0.984
Age of onset (years)	55.5 (47–63.25)	57 (49–61)	0.671
Disease duration (years)	9.5 (5.75–13.25)	9 (7–12.25)	0.794
LEDD	0.375 (0.297–0.453)	0.375 (0.312–0.375)	0.506
MDS-UPDRS III-11	2 (1–4)	2 (1.75–3.25)	0.724
NFOGQ	25 (15.75–26.25)	24 (20–27)	0.855
FOG type			0.974
Levodopa-responsive FOG	9 (90%)	52 (89.7%)	
Levodopa-resistant FOG.	1 (10%)	6 (10.3)	
Improvement rate	0.52 ± 0.17	0.53 ± 0.17	0.754
MDS-UPDRS I	14.6 ± 5.97	13.62 ± 5.72	0.656
MDS-UPDRS II	17 ± 4.72	22 ± 8.51	0.056
MDS-UPDRS III	62 ± 10.76	61 ± 16.30	0.263
MDS-UPDRS IV	6.4 ± 3.66	8.6 ± 3.82	0.881
BBS	40.5 (32–47.5)	41 (29–47)	0.768
Leg rigidity	2.5 (2.375–3)	2 (1.5–2.5)	0.025
Leg activity	2.52 ± 0.85	2.38 ± 0.90	0.580
Leg tremor	0.75 (0–2.125)	1 (0–1.625)	0.766
Balance	3 (1.75–3)	3 (2–3)	0.862
H-Y score	3 (2.5–3)	3 (2.5–3)	0.744
MOCA	20.4 ± 2.99	22.4 ± 4.16	0.208
MMSE	27.5 (24–29)	28 (25.75–29)	0.420
HAMA	7 (3.75–12.25)	7 (4–11)	0.931
HAMD	9.5 (6.5–11.75)	9 (5–13.25)	0.645
ESS	2 (1–4.5)	3 (0.75–6.25)	0.524
PSQI	96.4 ± 34.76	106.78 ± 27.32	0.284
RBD-HK	13.5 (5.5–29)	24 (10–38.75)	0.203
NMSS	41 (22.25–78)	51.5 (34.25–66.75)	0.368
UDysRS	8.5 (0–19.25)	16.5 (0–26.25)	0.433
On UDysRS			0.539
No	5 (50%)	23 (39.7%)	
Yes	5 (50%)	35 (60.3%)	
Off UDysRS			0.601
No	7 (70%)	45 (77.6%)	
Yes	3 (30%)	13 (22.4%)	

LEDD, Levodopa-equivalent daily dose; MDS, Movement Disorder Society; UPDRS, Unified Parkinson’s Disease Rating Scale; NFOGO, New Freezing of Gait Questionnaire; BBS, Berg Balance Scale; HY-stage scale, Hoehn and Yahr stage scale; UDysRS, Unified Dyskinesia Rating Scale; MoCA, Montreal Cognitive Assessment; MMMSE, Mini-Mental State Examination; HAMA, Hamilton Anxiety Scale; HAMD, Hamilton Depression Scale; ESS, Epworth Sleepiness Scale; PSQI, Pittsburgh Sleep Quality Index; RBD-HK, Rapid Eye Movement Sleep Disorder Questionnaire - Hong Kong edition; NMSS, Non-Motor Symptoms Scale.

### LASSO-logistic regression analysis screened the Most valuable variables

**Figure 1:**
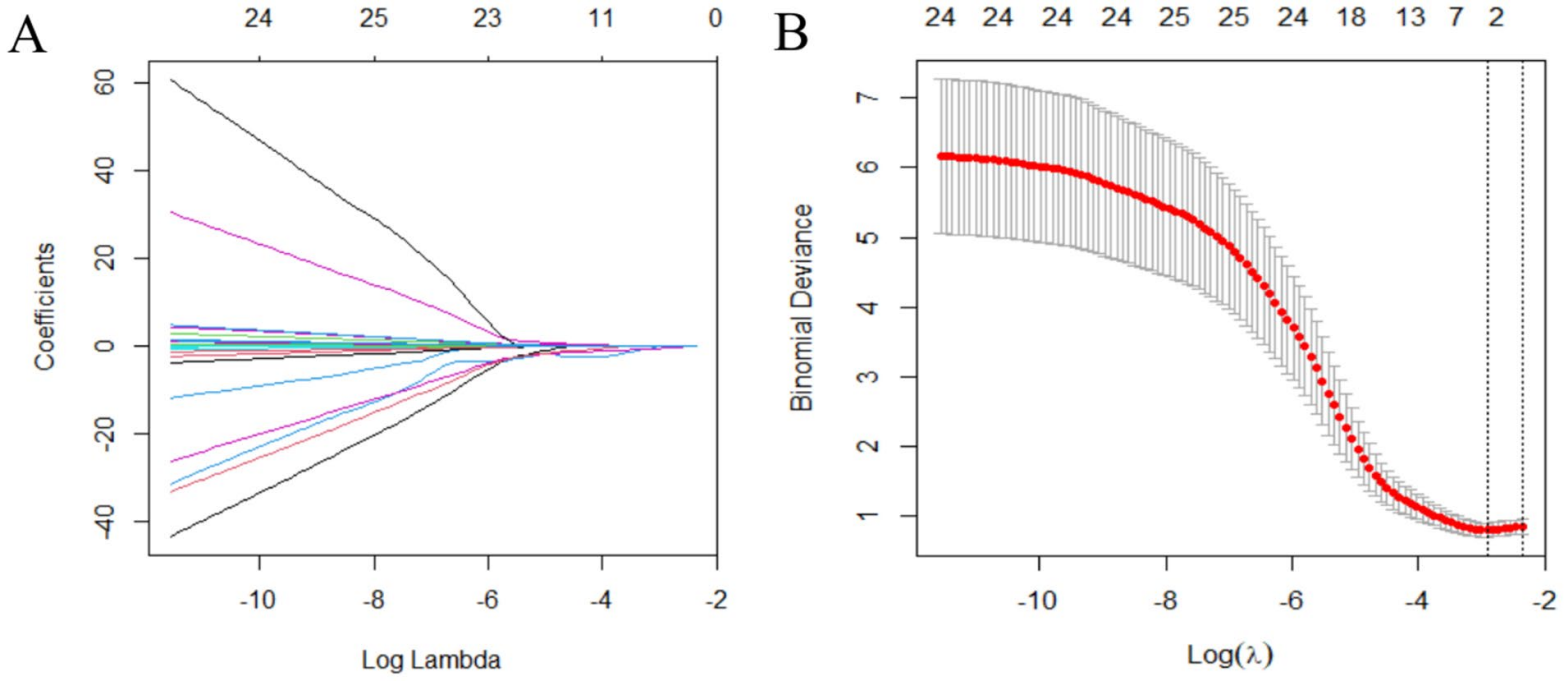
Variable selection using the LASSO binary logistic regression model. **(A)** LASSO coefficients of 30 variables. The optimal penalization coefficient (lambda) is identified in the LASSO model where optimal *λ* results in five features with non-zero coefficients. **(B)** The partial likelihood deviance (binomial deviance) curve is plotted against log(lambda). The left vertical line is dotted at the minimum criteria of λ, and the right vertical line is dotted at one standard error of the minimum λ. LASSO, least absolute shrinkage and selection operator.

Variables were selected using the LASSO binary logistic regression model in the training set. Optimal parameter selection (*λ*.min = 0.05438572) in the LASSO model was performed using 5-fold cross-validation with the minimum criteria. The binomial deviance curve was plotted against log(λ). The analysis revealed five characteristics, which included MDS-UPDRS II, MDS-UPDRS IV, leg rigidity, MoCA, and MMSE, with non-zero coefficients in the LASSO regression, which were selected as independent predictors (Figure 1).

Through intergroup comparisons between the improvement and non-improvement groups, a significant difference was found in leg rigidity (*p* = 0.025 < 0.05), suggesting that the severity of preoperative leg rigidity may be an important predictor of freezing of gait (FOG) improvement following STN-DBS. The multivariate logistic regression analysis further confirmed that leg rigidity is an independent predictor of postoperative FOG improvement (*p* = 0.028 < 0.05). Nonetheless, there was no significant difference in MDS-UPDRS II between the groups in the intergroup comparison (*p* = 0.056), but the multivariate logistic regression analysis revealed that the MDS-UPDRS II score was the strongest influencing factor (*p* = 0.026 < 0.05). (Table 2).

**Table 2:** Multivariate logistic regression analysis of STN-DBS effects on FOG in PD.

	Coefficient (β)	*Z*-value	*P*-value	OR	95% CI
Intercept	−1.0043	−0.26	0.7930	0.366	
MDS-UPDRS II	0.159	2.23	0.026	1.172	1.019–1.349
MDS-UPDRS IV	0.030	0.25	0.806	1.031	0.81–1.311
Leg rigidity	−1.726	−2.20	0.028	0.178	0.038–0.827
MoCA	0.121	0.98	0.329	1.128	0.886–1.438
MMSE	0.036	0.25	0.801	1.037	0.782–1.376

STN-DBS, Subthalamic nucleus deep brain stimulation; FOG, freezing of gait; MDS, Movement Disorder Society; UPDRS, Unified Parkinson’s Disease Rating Scale; MoCA, Montreal Cognitive Assessment; MMSE, Mini-Mental State Examination; OR, odds ratio; CI, confidence interval.

### Construction of the predictive nomogram

LASSO-logistic regression analysis identified the factors related to FOG symptom improvement in patients with PD after STN-DBS, which were then used to construct a prediction model. The prediction model formula is as follows:


$$Y=\beta_{0}+\beta_{1}X_{1}+\beta_{2}X_{2}+\beta_{3}X_{3}+\beta_{4}X_{4}+\beta_{5}X_{5}$$

Where 𝑌 is the dependent variable, *X*_1_, *X*_2_, *X*_3_, *X*_4_ and *X*_5_ are independent variables, *β*_0_ is the intercept, and *β*_1_, *β*_2_, *β*_3_, *β*_4_ and *β*_5_ are coefficients.

**Figure 2:**
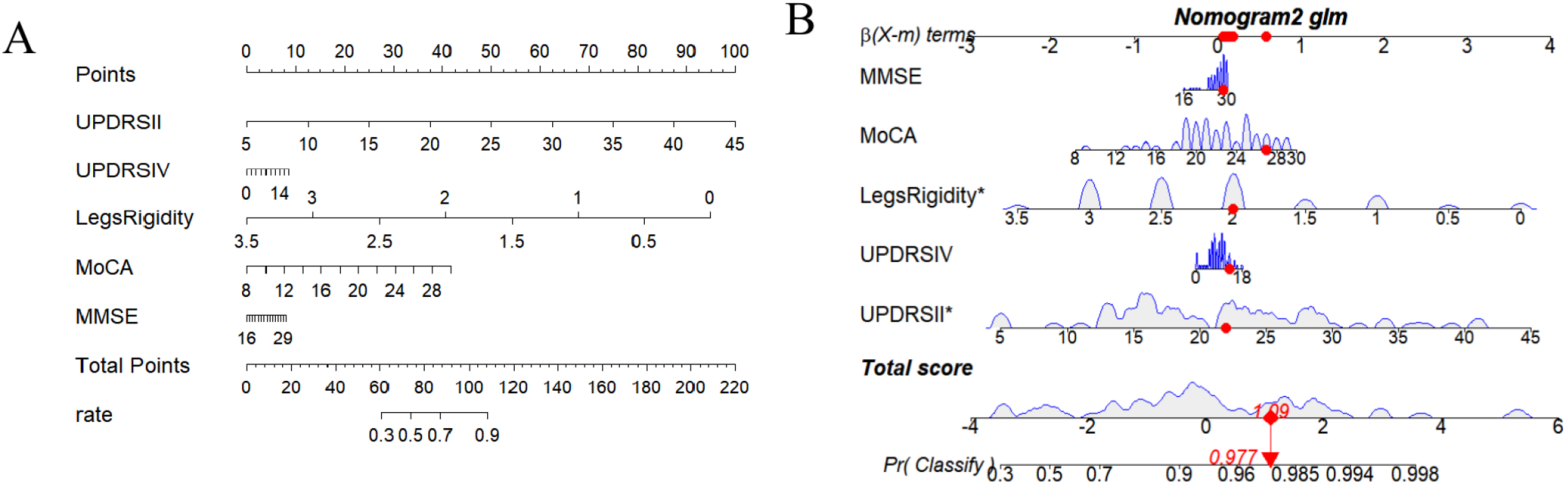
**(A)** Nomogram including MDS-UPDRS II, MDS-UPDRS IV, Legs rigidity, MoCA, and MMSE. **(B)** One patient is randomly selected, and the probability of postoperative FOG symptom improvement in this patient is predicted based on the five characteristic indicators of the nomogram.

FOG symptoms in patients with PD after STN-DBS could be easily and visually predicted using the nomogram drawn from this model. Figure 2A One patient was randomly selected, and the probability of postoperative FOG symptom improvement was 97.7% (Figure 2B).

### Predictive model evaluation and comparison

**Figure 3:**
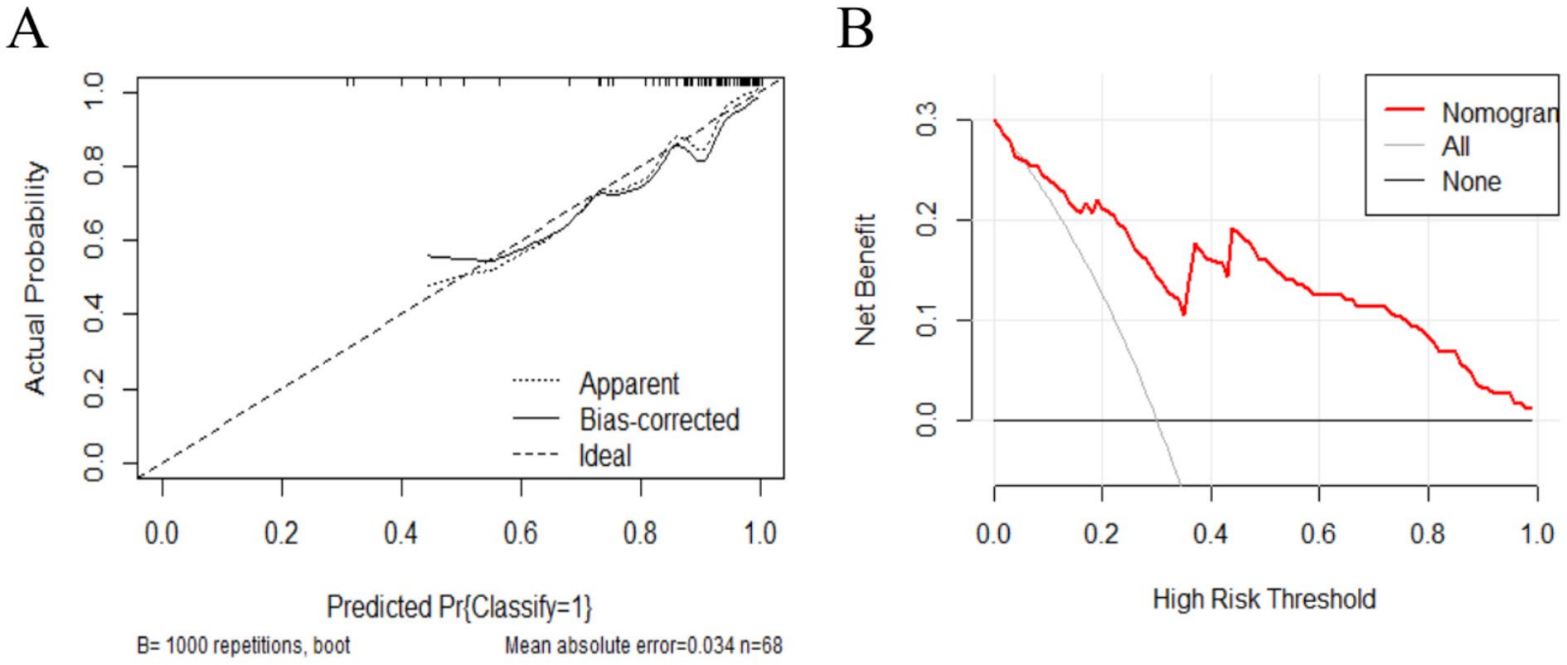
**(A)** The x-axis represents the predicted probability of FOG symptom improvement 1 year postoperatively, while the y-axis represents the actual probability. The diagonal line represents perfect prediction by an ideal model. The higher the ideal curve, the actual curve, and the calibration curve overlap, the better the model accuracy. **(B)** The x-axis represents the threshold probability, and the y-axis represents the net benefit. The solid line parallel to the x-axis represents patients who showed no postoperative improvement, the gray oblique solid line represents those who showed all improvement postoperatively, and the red curve represents the prediction model (nomogram) developed in the present study.

To assess the stability and reliability of the predictive model, we performed bootstrap resampling with 1,000 iterations. The bootstrap results demonstrated that the model’s performance was robust, with narrow confidence intervals, indicating high reliability in predicting the efficacy of bilateral STN-DBS for freezing of gait in Parkinson’s disease. Additionally, decision curve analysis (DCA) was performed to assess the clinical utility of the model (Figure 3A). The DCA curves demonstrated that the model provided a higher net benefit than the “improve all” or “improve none” strategies across a range of clinically relevant threshold probabilities, highlighting its potential value in guiding clinical decisions for bilateral STN-DBS in patients with freezing of gait (Figure 3B).

**Figure 4:**
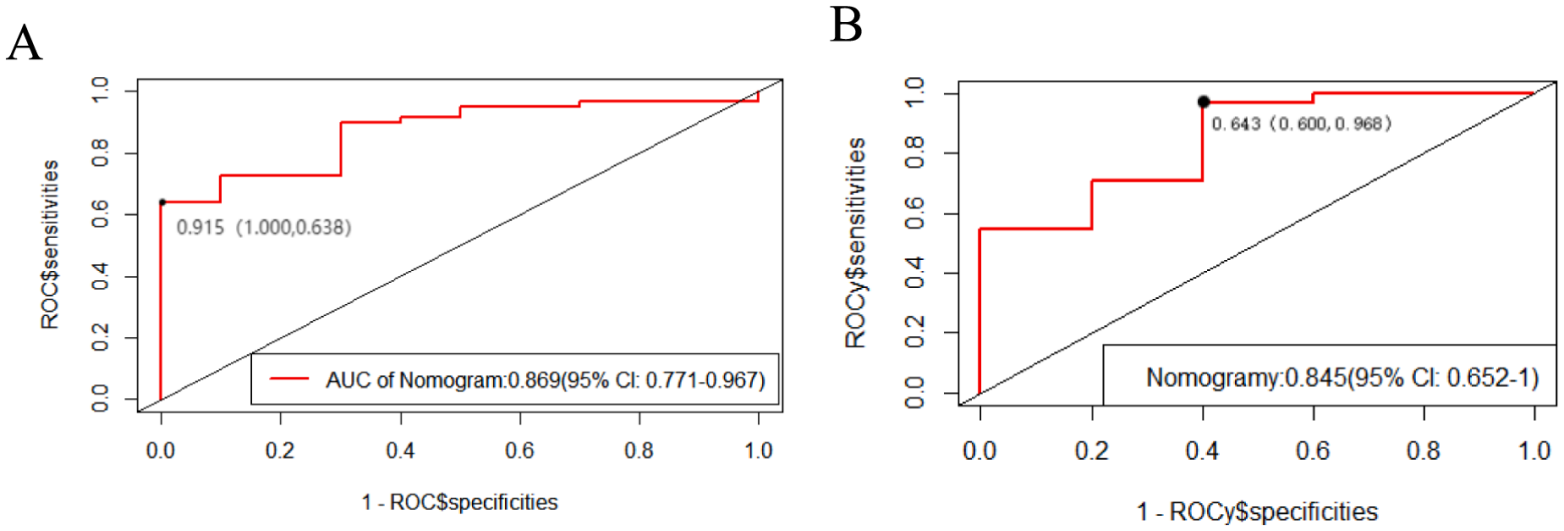
The area under the ROC curve is used to determine the predictive ability of the prediction model. The x-axis represents the false positive rate predicted by the model; while the y-axis represents the true positive rate predicted by the model. The red curve represents the performance of the nomogram. **(A)** The performance of the model in the training set. **(B)** The performance of the model in the validation set.

We plotted ROC curves for the LASSO model in the training set and validated this predictive model using the validation set. The area under the ROC curve is used to determine the predictive ability of the prediction model to distinguish outcomes. The AUC of the nomogram in the training set was 0.869 (95% confidence interval [CI]: 0.771–0.967). Furthermore, we revealed an AUC of 0.845 (95% CI: 0.6526–1) in the validation set. The AUC of both the training and validation sets highlighted the good predictive ability of the model. Therefore, the current model can accurately predict FOG symptom improvement in participants with PD receiving STN-DBS postoperatively (Figure 4).

## Discussion

FOG is a major disabling symptom that significantly affects the daily lives of patients with PD. The currently available treatment options for FOG include pharmacological treatments (Gao et al., 2020), surgical treatments, and physiotherapy (Gao et al., 2020). FOG tends to show a poor response to pharmacological treatments. Therefore, surgical treatments have become an indispensable approach to managing FOG. Surgical treatments, including DBS and spinal cord stimulation (SCS), have been reported to alleviate the symptoms of FOG (Opova et al., 2023). Among these symptoms, STN-DBS is one of the primary surgical treatments for FOG (Xie et al., 2015).

Based on the prediction model, we established that MDS-UPDRS II, MDS-UPDRS IV, leg rigidity, MoCA, and MMSE scores were the influencing factors for the improvement of FOG postoperatively. This is the most comprehensive study for developing and validating a prediction model based on the influence of STN-DBS on FOG in patients with PD. We analyzed the general characteristics and the scores of all preoperative assessment items in patients with PD when investigating the effect of STN-DBS on FOG, in addition to the individual scores on the UPDRS III, which may influence FOG, such as leg rigidity, leg tremor, and balance (Bloem et al., 2004; Nieuwboer and Giladi, 2008; Plotnik et al., 2009). The preoperative evaluation of the participants revealed that those with poor individual scores were more likely to have FOG symptoms or have worse FOG improvement. Previous studies on the effect of STN-DBS on FOG have only analyzed the general characteristics and common influencing scores preoperatively in patients with PD (Vercruysse et al., 2014; Schlenstedt et al., 2017; Barbe et al., 2020).

The MDS-UPDRS II score indicates the activities of daily living in PD patients. This study revealed that higher pre-operative UPDRS II scores were associated with more severe FOG symptoms in the Med-off/Stim-on state 1 year postoperatively. This finding indicates that poorer daily living ability before surgery is associated with greater postoperative improvement in FOG. Studies by Vercruysse et al. (2014), Schlenstedt et al. (2017), Zhang et al. (2021), Akturk et al. (2021), and Barbe et al. (2020) that investigated clinical factors related to FOG in PD also found that UPDRS II significantly influences the occurrence of FOG. They proposed that patients with poor daily living abilities were more likely to develop FOG, possibly due to the cumulative impact of motor and non-motor symptoms on their functional capacity. Combined with the results of our study, patients with poor daily living abilities may have more frequent or severe FOG symptoms at baseline. This phenomenon may be attributed to the greater improvement in patients with more severe symptoms, as well as the potential for STN-DBS to address both motor and non-motor aspects of PD that contribute to FOG. Our study, combined with existing literature, highlights the need for personalized treatment approaches that consider the mutual influence between activities of daily living, FOG severity, and other clinical factors in PD patients undergoing STN-DBS. Therefore, it is essential to optimize patient selection and postoperative management for maximal therapeutic benefit.

Our study revealed that the higher the preoperative leg rigidity score, the worse the postoperative FOG symptom improvement; this finding is consistent with several previous studies. For example, Fasano et al. (2012) found that patients with more severe preoperative motor symptoms, including rigidity, had a poorer response to STN-DBS, particularly in terms of axial symptoms such as FOG (Fasano et al., 2012). Additionally, Zibetti et al. (2011) demonstrated that the severity of preoperative leg rigidity was negatively correlated with gait improvement after STN-DBS. Wichmann and DeLong (2016) proposed that leg rigidity is primarily associated with abnormal activity within the basal ganglia-thalamocortical loop. Specifically, leg rigidity may arise from hyperactivity of the indirect pathway, leading to enhanced inhibitory motor signals that restrict the range and coordination of lower limb movements. STN-DBS modulates the activity of the subthalamic nucleus (STN), partially restoring the balance of the basal ganglia circuitry and thereby improving motor symptoms. However, in patients with more severe preoperative leg rigidity, the dysfunction of the basal ganglia-thalamocortical circuitry may have progressed to a degree that is less amenable to complete reversal through STN-DBS (Wichmann and DeLong, 2016). In combination with our study, for PD-FOG patients with more severe rigidity, STN-DBS is difficult to improve their FOG symptoms. This finding suggests that future STN-DBS treatment plans for FOG should consider patients’ leg rigidity symptoms to achieve personalized therapeutic strategies.

MMSE and MoCA are used to evaluate cognition in patients with PD. Our study revealed that the higher the preoperative cognitive score, the greater the postoperative FOG improvement. Previous studies (Scholl et al., 2021; Gan et al., 2023) have indicated that FOG severity is closely associated with impairments in executive function, attention, and visuospatial abilities, which are commonly affected in PD patients with cognitive decline. In addition, another study (Aybek et al., 2007) revealed that DBS surgery resulted in cognitive decline. Combined with the results of our study, these findings highlight the importance of carefully evaluating cognitive function before considering STN-DBS for the treatment of FOG in PD patients. Patients with poor preoperative cognitive function may not only experience limited improvement in FOG but also face a higher risk of postoperative cognitive decline. Therefore, STN-DBS surgery to improve FOG symptoms may not be advisable for patients with significant cognitive impairment.

MDS-UPDRS IV includes levodopa-induced dyskinesia, fluctuations in the on/off phenomenon, and other complications of levodopa therapy. This study revealed that a higher preoperative UPDRS IV score was associated with greater improvement in postoperative FOG symptoms. This finding was consistent with the results of a study by Vercruysse et al. (2014), which identified UPDRS IV as a clinical predictor of postoperative FOG symptom improvement. Additionally, Vercruysse et al. (2014) believed that FOG with more pronounced on/off fluctuations after consuming levodopa had a higher likelihood of having no-FOG postoperatively. Therefore, patients with a greater response to levodopa have better postoperative FOG symptom improvement.

The majority of studies (Vercruysse et al., 2014; Ferraye et al., 2008; Kleiner-Fisman et al., 2003; Schlenstedt et al., 2017) support the finding that the levodopa-responsive preoperative type is a predictor of postoperative motor symptom improvement, whereas the LEDD dose as a predictor remains controversial. Several studies (Ferraye et al., 2008; Niu et al., 2012) have revealed that LEDD dose is a predictor of postoperative FOG symptom improvement. However, in 2016, Schlenstedt et al. (2017) investigated the effect of STN-DBS on FOG among patients with PD through a meta-analysis and proposed the lack of correlation between the LEDD dose and postoperative FOG symptom improvement. Additionally, age, disease duration (Vercruysse et al., 2014), and neuropsychological function (Niu et al., 2012) were considered predictors of postoperative FOG symptom improvement. In contrast to the above results, our study revealed that MDS-UPDRS II and leg rigidity were the strongest influencing factors for postoperative FOG symptom improvement.

This study has some potential limitations. First, this was a retrospective study, and some clinical indicators with more vacancy values were eliminated, which may lead to unavoidable selection deviation. Second, the construction and verification of the prediction model were limited to PD patients with FOG who underwent STN-DBS surgery at Xuanwu Hospital of Capital Medical University, resulting in a small sample size. Data from patients with PD with FOG symptoms can be further collected in a multicenter and large-scale manner for model construction and verification. Third, the majority of studies confirmed that bilateral STN-DBS surgery improved FOG after 6 months or 1 year of follow-up (Kim et al., 2019); however, the long-term viability of these effects remains unclear. The follow-up period in this study was short, making the prediction of long-term efficacy uncertain. However, the prediction of short-term efficacy was relatively reliable. Fourth, the cohort included a large proportion of patients with levodopa-responsive freezing, thus caution should be exercised when extrapolating the data.

## Conclusion

This study developed a simple and effective model to predict the postoperative efficacy of bilateral STN-DBS surgery on FOG symptoms among PD patients. The obtained model can effectively assist clinicians in preoperatively assessing the therapeutic effects of STN-DBS on freezing of gait in Parkinson’s disease patients. Through personalized treatment planning, it aims to improve long-term quality of life and clinical outcomes. Based on the findings of this study, during preoperative evaluation, clinicians can focus on patients’ MDS-UPDRS II, MDS-UPDRS IV, leg rigidity, MoCA, and MMSE to predict the potential improvement of STN-DBS surgery on freezing of gait symptoms in Parkinson’s disease patients.

## Data Availability

Due to patient confidentiality agreements, the raw data containing substantial information beyond the scope of the results presented in this manuscript cannot be made publicly available.
